# Introducing Solid Foods to Infants in the Asia Pacific Region

**DOI:** 10.3390/nu6010276

**Published:** 2014-01-06

**Authors:** Madoka Inoue, Colin W. Binns

**Affiliations:** School of Public Health and Curtin Health Innovation Research Institute, Curtin University, GPO Box U1987 Perth, Western Australia 6845, Australia; E-Mail: C.Binns@curtin.edu.au

**Keywords:** complementary foods, infants, Asia pacific region, infant feeding practices

## Abstract

For infants’ optimal growth and development, the introduction of nutritionally suitable solid foods at the appropriate time is essential. However, less attention has been paid to this stage of infant life when compared with studies on breastfeeding initiation and duration. The practice of introducing solid foods, including the types of foods given to infants, in the Asia Pacific region was reviewed. In total nine studies using the same questionnaire on infant feeding practices were analysed to gain a better understanding of trends in the introduction of solid foods in this region. All studies showed less than optimal duration of exclusive breastfeeding indicating an earlier time of introduction of solid foods than recommended by the WHO. Most mothers commonly used rice or rice products as the first feed. In many studies, the timing of introducing solid foods was associated with breastfeeding duration. Compared with the Recommended Nutrient Intakes for infants aged above six months, rice/rice products are of lower energy density and have insufficient micronutrients unless they have been fortified. Although the timing of introducing solid foods to infants is important in terms of preventing later health problems, the quality of the foods should also be considered. Recommendations to improve the introduction of solid foods include measures to discourage prelacteal feeding, facilitating breastfeeding education and providing better information on healthier food choices for infants.

## 1. Introduction

Appropriate nutrient intake, in quantity, bioavailability, and timing in infancy are essential for optimal growth and development. Exclusive breastfeeding for six months and then the introduction of nutritious complimentary or solid foods, while breastfeeding continues, contributes to the prevention of acute and chronic diseases in early and later life [1]. Most reviews have concluded that “exclusive breastfeeding” for the first six months of life provides sufficient nutrients for infants for around six months, and then appropriate “complementary foods” should be introduced with continued breastfeeding, preferably until around two years of age or longer [2,3,4]. Both breastfed and infant formula fed infants should be introduced to safe and nutritious “complementary foods” at around six months to prevent retardation of growth and to minimize the risk of nutrient deficiencies [5].

“Complementary foods” are defined as foods other than breastmilk, infant formula or follow-on formula given to infants and these can be liquids, semi-liquids, and solids [6]. When these foods, particularly solid foods, are introduced to infants, textures should be changed as appropriate to the age of infants to give a variety of textural experiences. It is widely believed that foods with a pureed texture should be the first solid foods introduced [7]. “Solid foods” can be defined as non-drinkable food made by the food industry or by the family [8]. Complementary foods must also be nutritionally adequate and provide the bioavailable nutrients required, in combination with breastmilk, to meet all needs for growth and optimal health [4]. Since the nutrients in breastmilk are generally more bioavailable than from other sources, breastmilk remains an important component of nutrition after the introduction of solids. The term, “weaning” is often used to describe the infants who start taking solid foods [9] but “weaning” usually indicates a transition period or process from breastmilk or infant formula to solid foods [7]. It often refers to different events in different cultures and so it will not be used in this paper.

The timing of the introduction of solid foods is important because the early introduction of solid foods to infants by definition results in a shorter duration of “exclusive breastfeeding”, which in turn increases the risk of morbidity. Several studies have found that the early introduction of solid foods before six months of age has been associated with an increased risk of diarrheal disease or gastro-intestinal infection in infancy [10,11], food allergies [12], and overweight or higher Body Mass Index (BMI) in childhood [13]. The early introduction of solid foods may also change the composition of gastro-intestinal bacteria, the microbiome, which has implications for health [14,15,16]. In contrast, the late introduction of solid foods (after six months of age) predisposes to micronutrient deficiencies including iron and zinc status, which affect cognitive and neurological development [17,18] and may lead to other problems, including feeding difficulties [19].

In the most recent guidelines for infant feeding launched by the WHO/UNICEF in 2003, the introduction of solid foods to infants is recommended at six months of age (180 days) [20] but other international organizations recommendations may differ slightly from the WHO recommendation. The nutrition committee of the European Society for Pediatric Gastroenterology, Hepatology and Nutrition (ESPGHAN), recommended that the introduction of solid foods should not be commenced before 17 weeks of age and not later than 26 weeks of age. [21]. The American Academy of Paediatrics (AAP) stated that solid foods should not be commenced before six months of age [22]. Despite the WHO recommendations, many mothers have tended to introduce solid foods to their infants before six months. A cohort study of 401 mothers in Ireland found that the median age of introducing solid foods to infants was 16 weeks (interquartile range = 14–17.7), while 22.6% of the mothers introduced solid foods to their infants before 12 weeks postpartum [7]. In a British study (*n* = 604), the median age of introducing solid foods reported by the mothers was 15 weeks (interquartile range = 13–16), despite the national government recommendation to start solid foods for infants from six months of age [11].

In the Asia Pacific region, the recommended timing for the introduction of solid foods for infants varies between countries. For instance, in China, the Ministry of Health formerly recommended the introduction of solid foods to infants after four months of age (16 weeks), but more recently they have changed to six months of age [23]. In Australia, the National Health and Medical Research Council Infant Feeding Guidelines state that “solid foods” should be commenced at around six months of age [4]. The introduction of solid foods to infants is influenced by many cultural factors and traditional beliefs. For example, in Japan, a traditional ceremony is usually held at 100 days after birth and this ceremony was the time when mothers started to introduce additional liquid foods, including fruit juice and vegetable soup. Although the recent guidelines state that it is not necessary to provide any liquids other than breastmilk before six months, the ceremony still remains as a traditional custom in Japan. While changes in duration and exclusivity of breastfeeding have been extensively researched, less is known about how the patterns of infant feeding, including introducing solid foods, are changing and how these changes relate to differences in cultural practices between countries. The aim of this study was to review the timing of the introduction of solid foods in the Asia Pacific region by comparing and contrasting previous studies that used the same questionnaires and to describe the types of foods that are introduced.

## 2. Methods

### 2.1. Study Details and Descriptions of Sample Recruitments

Nine studies (five countries) undertaken by Curtin University, School of Public Health, of infant feeding practices conducted in the Asia Pacific region were reviewed including data on the first introduction of solid foods to infants. The recruitment process of the samples in each study has been published in detail elsewhere [24,25,26,27,28,29,30]. In Australia, the Perth Infant Feeding Study I (PIFS I) was undertaken to obtain information about infant feeding practices and provide information to assist in developing the Infant Feeding Guidelines and was repeated a decade later as the Perth Infant Feeding Study II (PIFS II). The questionnaires and methodology developed for these initial studies were then used in a similar way in other countries allowing for comparisons to be made. All of the Australian mothers in these studies were recruited in hospitals after birth. The Vietnamese mothers were recruited after giving birth in hospital, in community health centres, or at their home. In the two Chinese studies, all mothers were recruited at either hospitals or health centres as birthing at home is uncommon in this country. The mothers in the Zhejiang study were recruited from a city (Hangzhou), suburban, and rural areas. The Maldivian mothers were recruited at clinics associated with hospitals, the Japanese mothers were recruited at community health centres when they came for health examinations of their infants at 18 months of age and one study of mothers who have migrated to Australia used community samples recruited using “snowball” techniques. In the seven cohort studies the mothers were asked to complete an initial questionnaire on their infant feeding practices while in hospital and were then followed up at regular intervals for six months. Similar questionnaires were used in all of the studies and the three cross-sectional studies have been included in this review. The mothers in the Maldives study are representative of a conservative Islamic society, while the other countries are generally representative of Asia Pacific cultures.

### 2.2. Study Questionnaire

All reviewed studies used the same questionnaires on infant feeding practices and demographic details. Where necessary for cultural, translation, and ethical reasons several questions were modified. Specific areas of the questionnaire include:

- Demographics: maternal age, occupation, marital status, method of birth, parity, family income, husband’s/partner’s occupation, maternal smoking status, alcohol intake during lactation, infants’ birth weight
- Infant feeding practices: timing of feeding changes, intention to breastfeed, expressing breastmilk, breastfeeding problems, the reasons for ceasing breastfeeding, starting time of solid food and infant formula, and types of the first solid food given to infants.

### 2.3. Ethical Consideration

All of the studies were approved by the Human Research Ethics Committees of Curtin University and other relevant authorities. These included the local health authorities in Vietnam and Japan, and participating hospitals in China, Australia, and the Maldives. Confidentiality was assured and mothers were advised that their participation was voluntary and that they could withdraw at any time without prejudice.

## 3. Results

The details of each study including the study year, country, sample size, response rate, and methodology are shown in Table 1. Table 2 lists the sample characteristics reported by mothers in each study. In all studies, the majority of mothers were married and mothers in industrialized countries, particularly in cities, had more years of education than those in developing countries, but aboriginal mothers had the lowest education levels. In Australia, there are similar sample characteristics in maternal age in the PIFS I and II studies, while the Aboriginal mothers in PABS are younger. In both Chinese studies (Xinjiang, a remote area that is located in the Northwest, and Zhejiang, an industrialized province in Eastern China) most mothers were primiparous (75.8% and 88.6%, respectively) and there were higher rates of caesarian section (44.1% and 67.0%, respectively), than in the other studies. The Himeji study that was undertaken in the central part of Japan had the highest rate of low birth weight (8.4%) and unemployment in mothers (71.4%) among the studies.

**Table 1:** Details of the infant feeding studies in Asia Pacific region.

*	Authors	Data collection periods	Country	Study name	Sample size	Response rate (%)	Study method
1	Binns *et al.*	1992/93	Australia	Perth Infant Feeding Study I (PIFS I)	556	77	cohort
2	Binns *et al.*	2001/02	Australia	Perth Aboriginal Breastfeeding Study (PABS)	425	93	cohort
3	Duong *et al.*	2002	Viet Nam	Rural Viet Nam Infant Feeding Study	463	96	cohort
4	Binns *et al.*	2002/03	Australia	Perth Infant Feeding Study Mark II (PIFS II)	587	68	cohort
5	Xu *et al.*	2002/03	China	Xinjiang Infant Feeding Study	1219	97	cohort
6	Abdulraheem *et al.*	2004	Maldives	Maldives Infant Feeding Study	251	81	cross-sectional
7	Qiu *et al.*	2004/05	China	Zhejiang (Hangzhou) Infant Feeding Study	1520	96	cohort
8	Li *et al.*	2002	Australia	Chinese Infant Feeding Study living in Perth	506	95	cross-sectional
9	Inoue *et al.*	2007	Japan	Himeji Infant Feeding Study	1612	69	cross-sectional

* Sources: Study1: [31]; 2: [32]; 3: [29]; 4: [27]; 5: [28]; 6: [33]; 7: [34]; 8: [26]; 9: [25].

**Table 2:** The sample characteristics reported by mothers in each study.

Variables	Study 1 *	2	3	4	5	6	7	8	9
	*n* (%)	*n* (%)	*n* (%)	*n* (%)	*n* (%)	*n* (%)	*n* (%)	*n* (%)	*n* (%)
**Age (year)**									
<25	163 (29.3)	300 (70.6)	26.4 (4.97) (mean ± SD **)	154 (26.2)	184 (15.1)	198 (78.8) (below 30)	358 (23.6)	33 (6.5) (below 30)	67 (4.2)
25–29	193 (34.7)	80 (18.8)		170 (29.0)	544 (44.6)		800 (52.6)		170 (29.0)
30–34	135 (24.3)	41 (9.6) (above 30)		178 (30.3)	307 (25.2)	53 (20.4) (above 30)	338 (22.2)	473 (93.5) (above 30)	722 (44.8)
35≤	60 (10.8)			84 (14.3)	66 (5.4)				411 (25.5)
No response	6 (1.0)	4 (0.9)		1 (0.2)	118 (9.7)	0 (0.0)	24 (1.6)	0 (0.0)	61 (3.8)
**Marital status**									
Married/Defacto		370 (87.1)		540 (92.0)		239 (95.2)	1518 (99.9)	498 (98.2)	1535 (95.2)
Others	N/A	52 (12.2)	N/A	47 (8.0)	N/A	12 (3.2)	2 (0.1)	8 (1.8)	46 (3.1)
No response		53 (0.7)		0 (0.0)		0 (0.0)	0 (0.0)	0 (0.0)	28 (1.7)
<12	292 (52.5)	385 (90.8)	(85.5)	249 (43.2)	781 (64.1)	222 (88.5)	915 (60.5)	5 (1.0)	N/A ***
≥12	248 (44.6)	34 (8.0)	(14.5)	328 (56.8)	355 (29.1)	29 (10.4)	599 (39.6)	501 (99.0)	
No response	16 (2.9)	6 (1.2)	0 (0.0)	0 (0.0)	83 (6.8)	0 (0.0)	2 (0.1)	0 (0.0)	
**Parity**									
Primiparous	170 (30.6)	287 (66.8)		216 (36.8)	924 (75.8)		1347 (88.6)		780 (44.0)
Multiparous	383 (68.9)	107 (25.2)	N/A	371 (63.2)	199 (16.3)	N/A	163 (10.7)	N/A	899 (55.7)
No response	3 (0.5)	31 (7.3)		0 (0.0)	96 (7.7)		10 (0.7)		5 (0.3)
**Method of** **birth**									
Vaginal	454 (81.7)	214 (50.4)	428 (92.4)	411 (70.0)	602 (49.4)		495 (32.6)		1370 (85.0)
Caesarean	97 (17.4)	210 (49.4)	29 (6.3)	171 (29.1)	537 (44.1)	N/A	1019 (67.0)	N/A	240 (14.9)
No response	5 (0.9)	1 (0.2)	6 (1.3)	5 (0.9)	80 (6.6)		6 (0.4)		2 (0.1)
**Birth weight of infants**									
<2500 g	25 (4.5)	96 (22.6)	(3.0)	13 (2.2)	37 (3.0)		27 (1.8)		135 (8.4)
≥2500 g	531 (95.5)	329 (77.4)	(97.0)	566 (96.4)	1131 (92.8)	N/A	1479 (97.3)	N/A	1368 (84.9)
No response	0 (0.0)	0 (0.0)	0 (0.0)	8 (1.4)	51 (4.2)		14 (0.9)		109 (6.8)
**Maternal occupation**									
Unemployed			0 (0.0)	64 (10.9)	500 (41.0)	112 (44.6)	221 (14.5)	270 (53.4)	1151 (71.4)
Employed	N/A	N/A	463 (100.0)	512 (87.2)	644 (52.8)	139 (55.4)	1255 (82.6)	236 (46.6)	222 (26.2)
No response			0 (0.0)	11 (1.9)	75 (6.2)	0 (0.0)	44 (2.9)	0 (0.0)	39 (2.4)
**Partner’s/husband’s occupation**									
Unemployed		315 (74.1)	0 (0.0)			12 (4.8)	34 (2.2)		0 (0.0)
Employed	N/A	93 (21.9)	460 (99.4)	N/A	N/A	239 (95.2)	1420 (93.4)	N/A	1492 (92.6)
No response		17 (4.0)	3 (0.6)				58 (4.4)		120 (7.4)
**Maternal smoking status**									
Yes		299 (69.2)		196 (33.3)					192 (11.9)
No	N/A	126 (29.2)	N/A	321 (54.7)	N/A	N/A	N/A	N/A	1390 (86.2)
No response		6 (1.6)		70 (12.0)					30 (1.9)
**Partner’s/husband’s smoking status**									
Yes		64 (46.0) ^ψ^			705 (57.8)				786 (48.4) ^#^
No	N/A	75 (54.0) ^ψ^	N/A	N/A	383 (31.4)	N/A	N/A	N/A	712 (44.2)
No response		0 (0.0)			131 (10.8)				114 (7.1)

Source: 1: [31]; 2: [32]; 3: [29]; 4: [27]; 5: [28]; 6: [33]; 7: [34]; 8: [26]; 9: [25]; * Study numbers and sources are the same as above Table 1; ** SD = Standard Deviation; *** N/A = Not applicable as the ethics issues arisen; ^#^ including other family members; ^ψ^ *n*= 139.

Table 3 presents the median age of introducing solid foods to infants in the Asia Pacific region. Zhejiang is the earliest at 3.8 months, while Maldives and Japan were 5.5 months of age. In Vietnam, some mothers (4.8%) introduced solid foods to their infants as early as one week postpartum while the median age was approximately 4 months. Japanese mothers residing in Perth introduced solid foods earlier than those who are living in Japan. For Australian mothers the timing of introducing solid food to their infants changed over the decade between PIFS I and PIFS II with an increase in the mean age from 4.0 to 4.4 months. The most common first solid foods given to infants are rice or rice products in Asia Pacific region (Table 4) except in the Maldives where their traditional food which is made with wheat flour and fish, and Chinese migrants to Australia (egg-yolk). It is also interesting to note that over 40% of Vietnamese mothers used monosodium glutamate in the preparation of solid foods for infants [8].

**Table 3:** The median age of the first introducing solid foods (in months) by the studies.

*	Study name	Median age (SD) **
1	Perth Infant Feeding Study I (PIFS I)	4.0
2	Perth Aboriginal Breastfeeding Study (PABS)	4.7
3	Rural Viet Nam Infant Feeding Study	4.0
4	Perth Infant Feeding Study Mark II (PIFS II)	4.4
5	Xinjiang Infant Feeding Study	4.0
6	Maldives Infant Feeding Study	5.5 (2.0)
7	Zhejiang (Hangzhou) Infant Feeding Study	3.8
8	Chinese Infant Feeding Study living in Perth	N/A ^#^
9	Himeji Infant Feeding Study	5.5 (1.1)

* Study numbers are the same as Table 1; ** SD = Standard Deviation; ^#^ N/A = Not available.

**Table 4:** The type of solid foods given to infants in the Asia Pacific region.

Study number *	Study location	The most popular food	The second popular food	The third popular food
1	Australia, 1992	Rice cereal (commercial)	Fruit gels, puree	Milk Custards Yoghurt
2	Australia (Aboriginal mothers)	Milk Custards Yoghurt	Rice cereal (commercial)	Commercial foods with meat
3	Viet Nam	Rice porridge	Rice-floured porridge	Meat and egg
4	Australia, 2002	Rice cereal	Fresh/processed fruits and vegetables	N/A
5	China (Xinjiang)	Rice paste	Rice porridge	Vegetable paste
6	Maldives	Maldivian food made with wheat flour and fish	Rice porridge	Processed food
7	China (Hangzhou)	Rice cereal	Rice porridge	Mashed egg, fish
8	Australia (Chinese migrants)	Egg-Yolk	Commercial infant food	Fruit
9	Japan ^#^	Rice gruel	Japanese noodles	Puree vegetables

N/A = Not Applicable; ^#^ Reference [35].

Associations between the timing of introducing solid foods and breastfeeding duration were explored in each study. In Australia, in the PIFS II study, mothers who introduced solids at or after 17 weeks had 11 weeks longer duration of breastfeeding than those who introduced solids before 17 weeks (*p* < 0.001). The Japanese study also found that the timing of the introduction of solid foods was associated with the duration of “any breastfeeding” until six months of age (OR = 1.21, 95% CI = 1.10–1.33). Among Chinese migrants to Australia, mothers introduced solid foods to their infants at similar times to other Australian infants, but this was delayed when compared with mothers in home countries. In Viet Nam, significant factors associated with delayed introduction of solid food at 24 weeks were “if mother was a farmer” (OR = 0.52, 95% CI = 0.18–0.95) and “completed secondary school” (OR = 0.28, 95% CI = 0.10–0.54), whose “husband was satisfied with the infant’s gender” (OR = 0.30, 95% CI = 0.17–0.53), her “mother-in-law preferred exclusive breastfeeding” (OR = 0.18, 95% CI = 0.04–0.75), or her ‘friends practised exclusive breastfeeding’ (OR = 0.41, 95% CI = 0.16–1.10).

## 4. Discussion

While the timing of introducing solid foods varies between countries, most infants in the Asia Pacific region were introduced to solids earlier than recommended by the WHO. The mean age of introducing solid foods to infants in China (Hangzhou) was 3.8 months, the earliest in these studies, while Japan and Maldives were 5.6 months, closest to the WHO recommended age. Moreover, some studies showed that the timing of the introduction of solid foods was related to not only breastfeeding duration but also maternal occupation, education background, surrounding environments including preferences of family or friends on infant feeding methods. While the timing of solid food introduction is important in reducing problems related to infant health and development, the WHO has also emphasized the importance of the quality of the foods. Solid foods given to infants are often of high volume, with low energy and nutrient density together with a low meal frequency [36]. Our review found that many countries in the Asia Pacific region used rice porridge/cereal (See Table 4) for infants’ first foods since rice is culturally believed to help with digestion. Although some countries, including Japan, excluded this question for ethical reasons, other reports still described that the most common first solid foods was rice gruel [35,37]. These rice products are often of low energy and micronutrient density, including iron, zinc and calcium. In a report by Dewey and Brown [36], the WHO/UNICEF documented that energy requirements from solid foods for infants aged 6–8 months should be 269 kcal per day (1125.5 kJ) and the infants would be able to obtain sufficient energy if they were fed at least three meals with a minimum energy density of 1.0 kcal (4.2 kJ)/g. However, rice porridge has only 37.8 kcal (158 kJ) per 100 g (0.378 kcal/g), a low energy food (See Table 5) [38]. While the WHO report recommended that infants aged 6–8 months, 9–11 months, and 12–24 months should be fed at least 2–3 times, 3–4 times, and 3–4 times per day respectively, this is only applicable when energy and nutrient density is appropriate for the infants age [39]. For infants who are fed rice porridge to meet their energy requirements following the WHO recommendations, they would have to be fed approximately seven times per day. Similarly, the supply of micronutrient composition in rice products is less than the recommended nutrient intakes (Table 6). Several studies have shown that breastfed infants have better absorption of micronutrients, including iron. However, after six months of age, the quantities of micronutrients in breastmilk become inadequate over time, particularly for iron [40,41]. As this happens to both breast and bottle fed infants, the quality and timing of introduction of solid foods is important in providing adequate micronutrient intakes. In both developed and less developed countries, poor choices of solid foods may lead to nutritional deficiencies. 

**Table 5:** Nutritional composition of rice porridge, rice cereal and egg yolk (value per 100 g).

Main nutrients	Rice porridge	Rice cereal *	Hard-boiled egg yolk
Energy, including dietary fibre (kJ)	158	1537	1450
Protein (g)	0.7	6.8	16.1
Fat (g)	0.1	1.1	31.7
Calcium (mg)	2	6	115
Iodine (ug)	0.8	3.7	127.7
Iron (mg)	0.08	15.5	4.8
Zinc (mg)	0.12	7.8	2.7
Riboflavin (B2) (mg)	0.002	1.9	0.42
Pyridoxine (B6) (mg)	0.01	0	0.33
Vitamin C (mg)	0	33	0
Folate, natural (µg)	1	70	177

Source: [38]. * Note = this products was added vitamins B1, B2, B3, C, folate, iron and zinc.

**Table 6:** Recommended nutrient intakes for infants aged 7–12months and 12–24 months.

Main nutrients	7–12 months	12–24 months
Protein(g/day)	NA	NA
Calcium (mg/day)	400	500
Iodine (µg/day)	90	90
Iron (mg/day)	0.93 ^#^	0.58 ^#^
Zinc (mg/day)	4.1 *	4.1 *
Riboflavin (B_2_) (mg/day)	0.4	0.5
Pyridoxine (B_6_) (mg/day)	0.3	0.5
Vitamin C (mg/day)	30	30
Folate, natural (µg/day)	80	150

Source: [42]; Note: * = Moderate bioavailability; ^#^ = 95th percentile absolute requirements.

In developing countries, the inappropriate introduction of solid foods at an early age may be reflected in the proportion of stunting and/or wasting in young children [43]. Breastfeeding and nutritious solid foods play key roles in promoting appropriate nutrition for their growth and development and thus the quality of solid foods need to be focused to reduce the prevalence of under nutrition or malnutrition.

A meta-analysis on the impact of nutritional interventions on infant survival, disease prevention, and stunting concluded that child stunting could be reduced by approximately one third, if nutritional interventions were provided to infants before 36 months of age [44]. It is important to emphasize appropriate nutritious solid foods given to infants at the appropriate time. Golden [45] estimated the Recommended Nutrient Intakes (RNIs) for children who are moderately malnourished, and suggested the importance of a balance in nutrients between the macro- and micro-nutrients. This study also recognised the importance of nutrient density in the developing world, as many of the earliest foods introduced to infants are high volume with a low nutrient density.

Although our study showed that Maldives almost reached the WHO recommended age of introducing solid foods (5.5 months), the stunting rate under five years old was still 19% between 2006 and 2010 [46]. A more recent study in the Maldives found that within the first seven days after birth approximately 39% and 16% of infants (*n* = 458) were fed honey and dates, respectively, suggesting that the earlier study may have underreported prelacteal and early life feeds [47]. These prelacteal and early infancy feeds were related to specific cultural beliefs, but may also have had detrimental effects on infant health and the incidence of stunting.

In Japan, the mean age for the introduction of solid foods is approximately 5.5 months, and prelacteal feeds are still common, in contrast to the WHO recommendations for exclusive breastfeeding. The first priority for mothers is to continue exclusive breastfeeding for the first six months of life and then introduce nutritious complementary foods, appropriate nutrition during the 6–24 months period is also critical for infants’ nutrition and development [2]. Parents should be provided with more detailed information about introducing solid foods, including the quantity, timing, and quality of the foods through breastfeeding education since nutritional status during the first two years of life is critical in terms of their lifelong physical growth and mental development [5].

There are several limitations to consider when drawing conclusions from this study. Although these studies used almost the same questionnaire on infant feeding practices and included WHO standard infant feeding definitions, the sample selection and sizes used mean that the results may not be representative of the whole of the country. Nevertheless, similar methodology used in each study means that the main conclusions of this review can be used for nutrition education. The principal finding of the review is that most countries do not achieve the WHO goal for the timing of the introduction of solid foods. Increased promotion of optimum infant feeding guidelines is needed, including guidance for the appropriate time and manner in which solid foods are intoduced. This is an important public health message for infant nutrition in the Asia Pacific region.

## 5. Conclusions

The review of previous observational studies using the same questionnaire on infant feeding practices in the Asia Pacific region has shown that many countries need further improvement in the timing and the quality of first feeds with solid foods. This should be in conjunction with promoting the optimal duration of exclusive breastfeeding. Rice and rice products are commonly used as the first foods in this region and are of low energy density. Without fortification they provide insufficient quantities of micronutrients. Education of not only the mothers, but also other family members, health professionals and the community should be provided in order to facilitate understanding about the importance of breastfeeding and the appropriate introduction of solid foods. Several strategies including a general prohibition of prelacteal feeding in hospitals (except in specific medical circumstances), a ban on distribution of free gifts of infant formula to mothers, and an expansion of the roles of midwives should be explored. Further studies on this topic are required for a better understanding and evaluation of growth and development, and will be able to contribute to the development of more effective strategies in pediatric nutrition in this region.
